# The metabolic cost of walking on an incline in the Peacock (*Pavo cristatus*)

**DOI:** 10.7717/peerj.987

**Published:** 2015-06-02

**Authors:** Holly Wilkinson, Nathan Thavarajah, Jonathan Codd

**Affiliations:** Faculty of Life Sciences, University of Manchester, Manchester, UK

**Keywords:** Incline, Cost of transport, Peacock, Energetics, Locomotion, Bird

## Abstract

Altering speed and moving on a gradient can affect an animal’s posture and gait, which in turn can change the energetic requirements of terrestrial locomotion. Here, the energetic and kinematic effects of locomoting on an incline were investigated in the Indian peacock, *Pavo cristatus*. The mass-specific metabolic rate of the Indian peacock was elevated on an incline, but this change was not dependent on the angle ascended and the cost of lifting remained similar between the two inclines (+5 and +7°). Interestingly, the Indian peacock had the highest efficiency when compared to any other previously studied avian biped, despite the presence of a large train. Duty factors were higher for birds moving on an incline, but there was no difference between +5 and +7°. Our results highlight the importance of investigating kinematic responses during energetic studies, as these may enable explanation of what is driving the underlying metabolic differences when moving on inclines. Further investigations are required to elucidate the underlying mechanical processes occurring during incline movement.

## Introduction

Locomotion is an integral part of every organism’s life, and requires a significant proportion of daily energy expenditure (Karasov, 1981; Pontzer & Wrangham, 2004). Understanding how locomotor activity influences the daily energy budget of an animal is important as it can provide insights into adaptations that have evolved to alleviate the costs of moving around (Carrier, 1987; Webb, 1984). Research into the adaptations that relate to locomotor performance has provided valuable insight into the overall costs of animal movement (Bramble & Carrier, 1983; Cavagna, Heglund & Taylor, 1977; Parker, Robbins & Hanley, 1984). Relatively few studies, however, have sought to investigate how locomotion up gradients may influence the overall energy budget.

Movement over non-level terrain is interesting as animals rarely move over consistently level surfaces, as natural terrain is uneven (Lees et al., 2013). Increases in speed usually account for elevations in the energetic cost of terrestrial locomotion. This increase occurs as when speed increases, the force required to move the COM must be generated in less time, which is achieved by recruiting a higher volume of muscle and/or faster-acting muscle fibres (Ellerby et al., 2003; Full, 1987; Kram & Taylor, 1990; Taylor, Schmidt-Nielsen & Raab, 1970). However, locomotion on an incline can also increase metabolic rate when compared to moving on level ground (Warncke, Bandholtz & Schultze-Motel, 1988; Wickler et al., 2000; Wunder & Morrison, 1974). The increased cost of incline locomotion has been explained by the additional mechanical workload required to move the centre of mass (CoM) against gravity (Roberts et al., 1997; Wickler et al., 2005). Increasing the gravitational potential energy of the CoM is achieved by either recruiting a larger volume of muscle to generate the required energy (Pierotti et al., 1989), or by increasing the active shortening, and therefore strain, of a smaller volume of muscle (Daley & Biewener, 2003; Gillis & Biewener, 2002).

In an attempt to unify the costs associated with incline locomotion, Taylor, Caldwell & Rowntree (1972) deduced the cost to lift one kilogram one metre vertically as $15.5~\mathrm{J}~{\mathrm{kg}}^{-1}~{\mathrm{m}}_{\mathrm{v}}^{-1}$ across four mammalian species. However, subsequent empirical investigations have failed to find a similar cost of lifting (CoL) across a range of animals (Cohen, Robbins & Davitt, 1978; Farley & Emshwiller, 1996; Parker, Robbins & Hanley, 1984). The mass specific cost of incline locomotion has been found to be lower in larger animals, with efficiencies of metabolic energy use generally increasing with body mass (*M_b_*) (Snyder & Carello, 2008; Tullis & Andrus, 2011). Disparity among studies investigating the relationship between *M_b_*, speed and angle of inclination remains, with the relationship between efficiency (defined as the conversion of metabolic energy into mechanical work) and gradient also following no distinct pattern (Cohen, Robbins & Davitt, 1978; Warncke, Bandholtz & Schultze-Motel, 1988).

In terms of the energetic costs of locomotion broad similarities in the kinematics of terrestrial vertebrate groups are observed, but subtle differences in posture and gait give a proposed explanation for the species-specific variation (Nudds, Codd & Sellers, 2009; Reilly, McElroy & Biknevicius, 2007). Changing from a walking gait to an aerial running gait, where the in-phase relationship between E_p_ + E_kv_ (the sum of the gravitational potential and vertical kinetic energies of the CoM, used to raise the body’s CoM) and E_kh_ (the kinetic energy of the CoM used to reaccelerate the body for forward motion) results in no pendular exchange between potential and kinetic energy, is associated with lower energy economy (Heglund et al., 1982). However, the aerial running gait does afford some metabolic savings by increasing elastic potential energy storage in the tendons (Taylor, 1985). The stored elastic potential energy can be used to increase E_p_ + E_kv_ and E_kh_ and reaccelerate the CoM (Cavagna, Heglund & Taylor, 1977; Rubenson et al., 2004). Interestingly, bipedal birds often exhibit a grounded running gait (McMahon, Valiant & Frederick, 1987; Nudds et al., 2010). During grounded running, duty factor (DF, showing the ratio of limb contact with the ground during a stride) remains above 0.5 (Alexander, 2004), but E_p_ + E_kv_ and E_kh_ are in-phase (Heglund et al., 1982; Rubenson et al., 2004). Maintaining a higher DF increases the time available to generate the force required to raise the CoM (Roberts et al., 1998). The long tendons of birds are also stretched at low forces, allowing adequate elastic potential energy storage despite the in-phase relationship between E_p_ + E_kv_ and E_kh_ (Gatesy & Biewener, 1991; Rubenson et al., 2004; Taylor, 1985). Regardless of the apparent advantages, the effect of grounded running on the overall metabolic cost of locomotion, on level or gradient ground, has yet to be elucidated.

Studies investigating the effects of incline locomotion on kinematic parameters have found differences in DF (Dutto et al., 2004), stride frequency (Gillis & Biewener, 2002) and *t*_stance_ (the relative amount of time a foot is on the ground during a stride (Hoyt, Wickler & Cogger, 2000)) between level and incline locomotion. Force generation during *t*_stance_ is a direct measure of the metabolic cost of locomotion, and stride frequency has been directly associated with energetic cost, yet relatively few studies have investigated the metabolic cost of incline locomotion with the associated kinematic changes (Heglund et al., 1982; Lees et al., 2013; Nudds & Codd, 2012). Because the rate of force production is the parameter of interest, the inverse of contact time (1/*t*_stance_) is used for analysis and gives a measure of force application (Kram & Taylor, 1990). Combining information about the energetic and kinematic responses to locomotion on changing gradients may therefore provide valuable insight into the species-specific differences observed.

Here we investigated the energetic and kinematic response to two grades of incline locomotion in the peacock. We hypothesise that the energetic cost of locomotion in the peacock is higher on an incline than on the level gradient, and that movement on gradients influences locomotor kinematics.

## Materials and Methods

### Animals

We conducted respirometry experiments on peacocks (*n* = 6) (*M_b_* = 4.58 ± 0.14 kg). The peacock, of the order Galliformes, is a large (4–6 kg), primarily terrestrial bird species (Harikrishnan, Vasudevan & Sivakumar, 2010). During the experimental period, the birds were housed together and were given *ad libitum* access to food and water. Birds were trained for 2 weeks and were not fasted prior to data collection. All birds were 4 years old.

### Respirometry

To measure oxygen consumption (*V*_O_2__) and carbon dioxide production (*V*_CO_2__), open flow indirect calorimetry was used (all equipment and computer programs Sable Systems International^®^, Las Vegas, Nevada, USA). The birds were exercised at randomized speeds up to their maximum sustainable speed. Birds were trained to walk inside a Perspex© box (volume 620 L) mounted on a treadmill (Professional Model, Fit Fur Life, Surrey, UK). Air was pulled through using a Flow-Kit 2000, at 450 L min^−1^ (FR). The excurrent flow was then subsampled at 0.1 L min^−1^ for gas analysis. Water vapour pressure (WVP) was recorded using an RH-300 and scrubbed from the air mixture using calcium chloride (2–6 mm granular; Merck, Darmstadt, Germany). The sample was then drawn through a CA-10 carbon dioxide analyser before CO_2_ was scrubbed using soda lime (2–5 mm granular, Sigma Aldrich, Steinheim, Germany). Finally, O_2_ concentration and barometric pressure (BP) were measured using an Oxilla II. Ambient air (scrubbed of H_2_O and CO_2_, as before) was simultaneously drawn through the second channel of the Oxilla II at 0.1 L min^−1^ by a separate pump (SS-3) to enable calculation of differential O_2_ concentration (ΔO_2_). Background CO_2_ was subtracted from the measurements to calculate differential CO_2_ concentration (ΔCO_2_). Outputs were recorded using a UI-2 and analysed using ExpeData^®^ Software. The accuracy of the system (±4%) was validated by N_2_ dilution tests (Fedak, Larry & Seeherman, 1981). Primary flow rates were adjusted to dry-corrected flow rates (FR_c_) to account for the H_2_O scrubbed from the air samples prior to gas measurement using Eq. (1): (all equations from (Lighton, 2008). *V*_O_2__ rates (Eq. (2)) were not corrected for CO_2_ (see Withers, 1977) as this has a negligible effect.

(1) \begin{eqnarray*} {\mathrm{FR}}_{\mathrm{c}}=\frac{\mathrm{FR}\cdot (B P-W V P)}{B P} \end{eqnarray*}

*V*_O_2__ was calculated using:

(2) \begin{eqnarray*} {V}_{{\mathrm{O}}_{2}}=\frac{{\mathrm{FR}}_{\mathrm{c}}(\Delta {\mathrm{O}}_{2})}{(1-0.2095)} \end{eqnarray*}

and *V*_CO_2__ using:

(3) \begin{eqnarray*} {V}_{{\mathrm{CO}}_{2}}=\frac{{\mathrm{FR}}_{\mathrm{c}}(\Delta {\mathrm{CO}}_{2})-(0.0004({V}_{{\mathrm{O}}_{2}}))}{(1-0004)}. \end{eqnarray*}

Metabolic power consumption (*P*_met_, W kg^−1^) was converted from *V*_O__2_, using the respiratory exchange ratio (RER: *V*_CO__2_: *V*_O__2_) and thermal equivalents taken from Brody (1945). Net–*P*_met_ was calculated by subtracting resting metabolic rate (RMR, W kg^−1^) from locomotor *P*_met_ (both from the same trial).

### Peacock trials

As previous studies on bird energetics have shown the energetic cost of locomotion to increase linearly with speed (*U*) (Lees et al., 2013; Nudds et al., 2010; Taylor, Heglund & Maloiy, 1982), three representative speeds (0.5, 0.75, 1.0 m s^−1^) were selected. The inclines chosen (0°, 5° and 7°) reflected the inclines used in previous studies on bipedal birds (Lees et al., 2013; Nudds & Codd, 2012). In each trial, bird and *U* were selected at random on an incline randomly chosen for the particular day (i.e., 0, +5 or +7°). Each bird was given a warm-up walk on the treadmill of 1-2 min at 0.5 m s^−1^ then allowed to rest until the oxygen trace stabilised (where fluctuations in the trace were <0.001%) and stayed constant for at least 2 min (Halsey et al., 2009; Nudds & Codd, 2012). The birds ran at each trial *U* until oxygen levels reached a plateau (∼5 min). At the completion of each trial the birds rested until a stable resting respiration rate was achieved (∼4 min). The mean O_2_ and CO_2_ concentrations were taken from the final 80 s of the respirometry trace for each *U* when the readings were stable. Resting gas concentrations were also taken from the last 80 s of the most stable recovery period.

### The metabolic cost of lifting

The CoL shows the cost of incline movement relative to the cost of moving on the level. The MCoT (the minimum amount of energy required to move one gram of animal one kilometre, J kg^−1^ m^−1^) was used to calculate the CoL. The MCoT values were the lowest values in a plot of the total cost of transport (calculated by dividing *P*_met_ by *U*) versus *U* (Nudds, Codd & Sellers, 2009). The metabolic cost of lifting one kilogram (kg) of body mass one metre (m) vertically $(\mathrm{J}~{\mathrm{kg}}^{-1}~{\mathrm{m}}_{\mathrm{v}}^{-1})$ was then determined using the following equation (Lees et al., 2013):

(4) \begin{eqnarray*} [({\mathrm{MCoT}}_{\mathrm{in}}-{\mathrm{MCoT}}_{\mathrm{h}})/\sin \theta ] \end{eqnarray*}

where MCoT_in_ and MCoT_h_ represent the MCoT on the incline and horizontal, respectively. The efficiency (%) of converting metabolic energy into mechanical energy for vertical work could then be estimated by dividing the mechanical work to lift one kilogram one meter vertically $(9.8~\mathrm{J}~{\mathrm{kg}}^{-1}~{\mathrm{m}}_{\mathrm{v}}^{-1})$ by the metabolic energy to lift 1 kg 1 m vertically (Tullis & Andrus, 2011).

### Kinematics

To determine the kinematic parameters of peacock locomotion, a high-speed camera (Sony HDR-XR520VE; Sony, Minato, Tokyo, Japan) was used at each *U* with a frame rate of 25 frames per second (fps) at 0.5 m s^−1^ and 100 fps at 0.75 m s^−1^ and 1 m s^−1^. At 0.5 m s^−1^ peacocks were filmed for ten strides. At 0.75 m s^−1^ and 1 m s^−1^ 3–4 strides per video. Peacocks 2D kinematics were filmed from a lateral view. Tracker v4.85 software (Open Source Physics) was used to quantify the footfall events of both level and incline locomotion (Lees et al., 2013). DF, swing time (Roberts et al., 1997), stride length (Gatesy & Biewener, 1991) and stride frequency (the rate of foot movement during a stride, *f*_stride_) were calculated (Lees et al., 2013).

As it is difficult to discern a change from walking to grounded running solely from relative limb support times (Gatesy & Biewener, 1991), estimates of the location of the CoM were made on four birds to calculate any gait changes depending on *U*. For the CoM trials, a marker was placed on the birds on the outside (left) wing. A high-speed video (100 fps) was taken at each *U*, ranging from 0.5 m s^−1^ to 1 m s^−1^. The videos were analysed in Tracker v4.85 software (Open Source Physics). The output was used to calculate the E_kh_, E_p_ + E_kv_ and the *E*_tot_ (the total of the E_kh_ and E_p_ + E_kv_) at each *U* on the level and at a 7° incline (Nudds et al., 2010).

### Statistical analysis

Sample sizes were chosen based on power analyses of our previous work. Differences in net-*P*_met_ as well as kinematic parameters, plotted against *U*, were tested using a linear mixed effects model (LME) with an accompanying Tukey post-hoc where necessary. Statistical results were derived from the minimum adequate model i.e., non-significant interaction terms were removed from the LME. The same individuals were sampled for all gradients, for these analyses individual was included as a random factor. A one-way ANOVA was performed on the log_10_ transformed (to normalise the data) MCoT values at 0.75 m s^−1^ to investigate if there was a significant effect of incline on MCoT. A one-way ANOVA was also performed on resting *P*_met_ to test if there was an effect of the incline treatment on resting metabolic rate. All statistical analyses were performed in R v3.1.1 (R Development Core Team, 2014). Experiments were performed under Home Office Project Licence (40/3549) held by JRC and with approval from the Animal Ethical Review Group of the University of Manchester.

## Results

### Energetics

**Figure 1:**
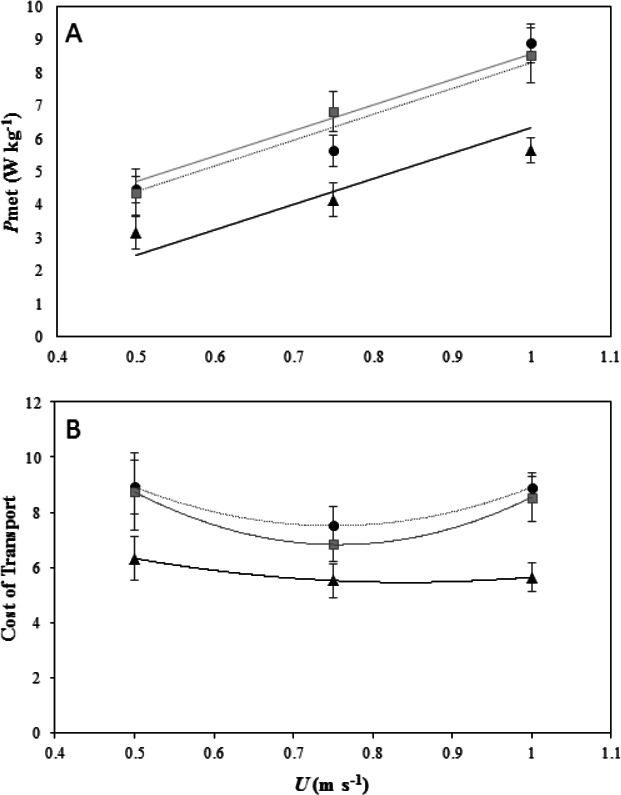
Energetics of locomotion for peacocks moving of different incline. (A) Net Mass-specific power consumption (*P*_met_, mean ± s.e.m) plotted against forward speed (*U*) on a level treadmill (black dotted line and black, triangle markers), on a 5° incline (black dotted line and black, circle markers), and on a 7° incline (grey solid line and grey, square markers). All 6 birds performed the first 2 speeds on the level and at each incline and 4 birds performed the top speed at the highest incline. The lines fitted through the data are from the LME model output and are *P*_met_ = − 1.40 + 7.74*U* for peacocks moving on a level gradient, *P*_met_ = 0.53 + 7.74*U* for peacocks moving on a 5° *incline and* *P*_met_ = 0.53 + 7.74*U for peacocks moving on a* 7° incline. (B) The minimum cost of transport (MCoT) plotted against forward speed (*U*) on a level treadmill (black dotted line and black, triangle markers) and at inclines of 5° *(black dotted line and black, circle markers) and* 7° (grey solid line and grey, square markers). The MCoT, used to calculate the cost of lifting (CoL), was found at 0.75 m s^−1^ in each gradient treatment.

Net-*P*_met_ increased linearly with increasing *U* at the same incremental rate for each gradient (Incline × *U*, *F*_2,39_ = 5.03, *P* = 0.08) and net-*P*_met_ increased with increasing gradient as indicated by differences between the intercepts of the slopes (Fig. 1A, LME: *U*, *F*_1,41_ = 84.48, *P* < 0.001; incline, *F*_2,41_ = 36.34, *P* < 0.001;). A Tukey post-hoc test indicated a significant difference at the 0.05 confidence level between net-*P*_met_ on the level and at each incline (level and 5°, *Z* = 5.19, *P* < 0.001; level and 7°, *Z* = 5.67, *P* < 0.001). No difference was found in net-*P*_met_ between the two inclines 5° and 7°(*Z* = − 0.73, *P* = 0.74). There was no significant effect of incline on resting *P*_met_ (ANOVA: *F*_2,15_ = 3.13, *P* = 0.07).

### Cost of lifting

The MCoT for each gradient was at 0.75 m s^−1^ (Fig. 1B). The MCoT was 5.52 J kg^−1^ m^−1^ on the level gradient, 7.51 J kg^−1^ m^−1^ on a 5° incline, and 6.8 J kg^−1^ m^−1^ on a 7° incline (see supplementary material Table S1). The MCoT was significantly affected by incline (ANOVA *F*_2,45_ =10.78, *P* < 0.001). A Tukey post-hoc test indicated a significant difference at the 0.05 confidence level between MCoT_in_ and MCoT_h_ at incline gradients of 5° (95% CI [−4.01, −1.14], *P* < 0.001) and 7° (95% CI [−3.57, −0.66], *P* = 0.003), but not between the two inclines 5° and 7° (95% CI [−1.91, 0.99], *P* = 0.73). From the MCoT values, the CoL was calculated as $22.84~\mathrm{J}~{\mathrm{kg}}^{-1}~{\mathrm{m}}_{\mathrm{v}}^{-1}$ at a 5° incline and $24.79~\mathrm{J}~{\mathrm{kg}}^{-1}~{\mathrm{m}}_{\mathrm{v}}^{-1}$ at a 7° incline, with efficiencies of 42.91% and 39.53%, respectively.

### Kinematics

**Figure 2:**
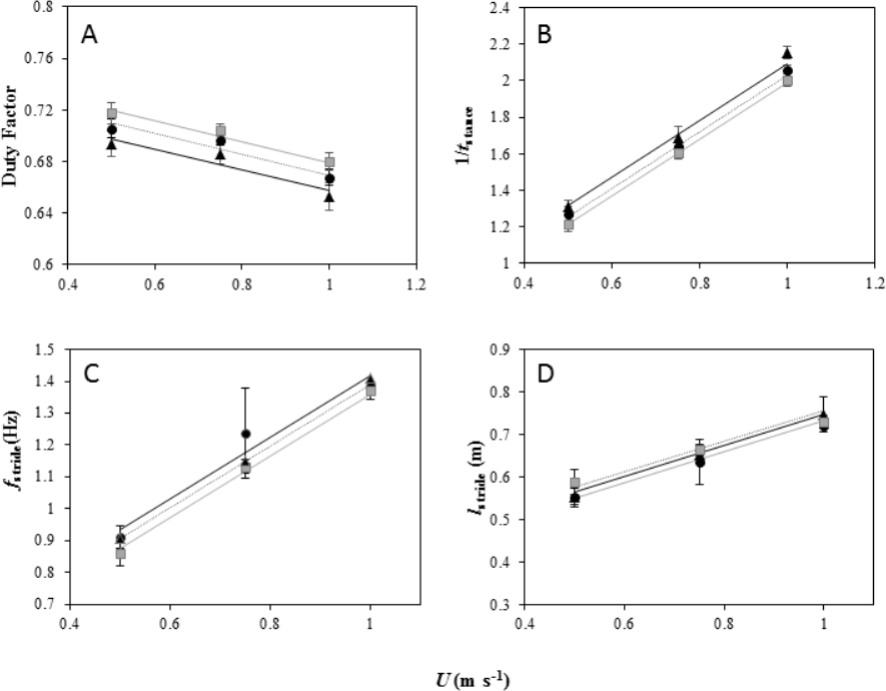
Foot kinematics of peacocks moving on a level treadmill (black dotted line and black, triangle markers) and at inclines of 5° (black dotted line and black circle markers) and 7° (grey solid line and grey, square markers). All 6 birds performed the first 2 speeds on the level and at each incline and 4 birds performed the top speed at the highest incline. (A) Duty factor (mean ± s.e.m) plotted against forward speed (*U*) for each gradient. The lines fitted through the data (from the LME model output) are *P*_met_ = 0.73 + − 0.08*U* for peacocks moving on a level gradient, *P*_met_ = 0.75 + − 0.08*U* for peacocks moving on a 5° incline and *P*_met_ = 0.75 + − 0.08*U for peacocks moving on a* 7° incline. (B) Inverse of contact time (1/*t*_stance_) plotted against forward speed (*U*) for each gradient. The lines from the model output are *P*_met_ = 0.53 + 1.55*U* for peacocks moving on a level gradient, *P*_met_ = 0.47 + 1.55*U* for peacocks moving on a 5° incline and *P*_met_ = 0.43 + 1.55*U for peacocks moving on a* 7° *incline*. (C) *f*_stride_ plotted against forward speed (*U*) for each gradient. The lines from the model output are *P*_met_ = 0.42 + 0.96*U* for peacocks moving on a level gradient, *P*_met_ = 0.44 + 0.96*U* for peacocks moving on a 5° incline and *P*_met_ = 0.39 + 0.96*U* for peacocks moving on a 7° incline. (D) *l*_stride_ plotted against forward speed (*U*) for each gradient. The lines from the model output are *P*_met_ = 0.38 + 0.36*U* for peacocks moving on a level gradient, *P*_met_ = 0.36 + 0.36*U* for peacocks moving on a 5° incline and *P*_met_ = 0.39 + 0.36*U for peacocks moving on a* 7° incline.

DF decreased linearly with increasing *U* at the same incremental rate for each gradient (Incline × *U*, *F*_2,39_ = 0.15, *P* = 0.93) and DF increased with increasing gradient as indicated by differences between the intercepts of the slopes (Fig. 2A, LME: *U*, *F*_1,41_ = 80.74, *P* < 0.001; Incline, *F*_2,41_ = 25.48, *P* < 0.001). A Tukey post-hoc test indicated a significant difference at the 0.05 confidence level between DF on the level and at each incline (level and 5°, *Z* = 2.94, *P* = 0.009; level and 7°, *Z* = 5.02, *P* < 0.001). No difference was found in DF between the two inclines 5°and 7°(*Z* = − 2.13, *P* = 0.08). DF remained above 0.5 in all treatments, which showed the birds did not reach an aerial running phase. The minimum DF recorded was 0.65 ± 0.02 at 1 m s^−1^ when walking on the level gradient.

1/*t*_stance_ increased linearly with increasing *U* at the same incremental rate for each gradient (*F*_2,39_ =1.93, *P* =0.38) and 1/*t*_stance_ decreased with increasing gradient as indicated by differences between the intercepts of the slopes (Fig. 2B, LME: *U*, *F*_1,41_ = 1813.6, *P* < 0.001; Incline, *F*_2,41_ = 35.37, *P* < 0.001). A Tukey post-hoc test indicated a significant difference at the 0.05 confidence level between 1/*t*_stance_ on the level and at each incline, as well as between the incline gradients (level and 5°, *Z* = − 3.55, *P* = 0.001; level and 7°, *Z* = − 5.9, *P* < 0.001; 5° and 7°, *Z* = 2.41, *P* = 0.04).

*f*_stride_ increased linearly with increasing *U* at the same incremental rate for each gradient (Incline × *U*, *F*_2,39_ = 0.15, *P* = 0.93) and there were no differences in *f*_stride_ between the gradients (Fig. 2C, LME: *U*, *F*_1,41_ = 129.61, *P* < 0.001; Incline, *F*_2,41_ = 1.87, *P* = 0.39. *l*_stride_ increased linearly with increasing *U* at the same incremental rate for each gradient (Incline × *U*, *F*_2,39_ = 1.5, *P* = 0.47) and there were no differences in *l*_stride_ between the gradients (Fig. 2D, LME: *U*, *F*_1,41_ = 98.89, *P* < 0.001; Incline, *F*_2,41_ = 1.87, *P* = 0.39).

*t*_swing_ decreased linearly with increasing *U* at the same incremental rate for each gradient (Incline × *U*, *F*_2,39_ = 0.004, *P* = 1.00) and there were no differences in *t*_swing_ between the gradients (LME: *U*, *F*_1,41_ = 110.89, *P* < 0.001; Incline, *F*_2,41_ = 1.68, *P* = 0.43).

On the level gradient, E_kh_ and E_p_ + E_kv_ were out of phase indicating that the peacocks were using a walking gait at each of the three speeds (0.5 m s^−1^, 0.75 m s^−1^, 1 m s^−1^). At a 7° incline, E_kh_ and E_p_ + E_kv_ were in-phase at 0.75–1.0 m s^−1^, illustrating that two of the four birds analysed were grounded running at 1 m s^−1^, with one of these birds grounded running at 0.75 m s^−1^. Overall, the CoM results suggest that peacocks may change from a walking to a grounded running gait at lower speeds on an incline than on the level.

## Discussion

The energetic cost of locomotion in the peacock is greater while moving on an incline gradient than on a level gradient, consistent with previous investigations in birds (Ellerby et al., 2003; Lees et al., 2013; Rubenson et al., 2006), reptiles (Farley & Emshwiller, 1996; Zani & Kram, 2008), mammals (Cohen, Robbins & Davitt, 1978; Eaton et al., 1995; Fancy & White, 1987) and some invertebrates (Full & Tullis, 1990; Tullis & Andrus, 2011). Energetic cost also increased linearly with *U* in each treatment, as in most studies, with only a few exceptions where the energetic cost of locomotion increases curvilinearly with *U* (Langman et al., 1995; Snyder & Farley, 2011; Steudel-Numbers & Wall-Scheffler, 2009; Wickler et al., 2005).

The elevated metabolic cost associated with incline locomotion has been attributed to increases in muscle activity required to raise the CoM against gravity, while accounting for the apparent reduction in elastic potential energy (Gabaldón, Nelson & Roberts, 2004; Roberts et al., 1997; Snyder & Farley, 2011). During incline movement, increased muscle shortening also raises metabolism as eccentric muscle contractions are replaced by more costly concentric muscle contractions (Daley & Biewener, 2003; Wickler et al., 2005). Concentric muscle contractions generate less tension than eccentric muscle contractions (Davies & Baknes, 1972; Proske & Morgan, 2001), resulting in the recruitment of a larger volume of muscle to generate the force required for incline locomotion (Gabaldón, Nelson & Roberts, 2008). Increased blood flow to stance phase muscles (Rubenson et al., 2006) and elevated stance phase muscle activity (Gabaldón, Nelson & Roberts, 2004) have also been found, which may further suggest that incline locomotion requires more positive muscular work than level movement.

We found no difference in the energetic cost of locomotion between the two incline gradients studied, although these differed only by 2 degrees. Previously, the energetic cost of locomotion has been shown to increase as incline rises (Eaton et al., 1995; Full, 1987; Raab, Eng & Waschler, 1976) or stay constant between inclines (Chassin et al., 1976; Lees et al., 2013; Lipp, Wolf & Lehmann, 2005), with no clear differences between bipedal, quadrupedal or hexapedal animals demonstrated. However, relating the energetic cost of locomotion to total force output is required to account for factors other than net mechanical work (e.g., isometric muscle contractions and muscle efficiency) influencing metabolic rate (Biewener, 1990; Roberts et al., 1997). Alterations in posture, gait, joint mechanics and footfall events can also affect the magnitude of force required for locomotion (Birn-Jeffery & Higham, 2014; Hesse et al., 2014; Lammers, Earls & Biknevicius, 2006; Reilly, McElroy & Biknevicius, 2007).

One of the most relevant investigations emphasised the importance of the rate of force application on the overall cost of transporting body weight. The comparative approach across a number of mammalian species found the larger the animal, the lower the rate of force application (due to a larger *l*_stride_ and lower *f*_stride_ allowing a larger *t*_stance_) and subsequently, the lower the transport costs per gram of body weight (Kram & Taylor, 1990). The effects of incline on the energetic cost of locomotion are less well understood; however, it is apparent that the kinematic results may provide a clearer understanding of the metabolic costs associated with incline locomotion.

In a number of investigations, few kinematic parameter changes are found between horizontal and incline locomotion (Eaton et al., 1995; Full & Tullis, 1990; Lees et al., 2013). In the present study, however, peacocks maintained higher DF’s on an incline than on the level (Nudds & Codd, 2012). The increase in DF, and the decrease in 1/*t*_stance_ found when comparing level and incline movement (Oldruitenborgh-Oosterbaan, Barneveld & Schamhardt, 1997), indicated that the increased cost of incline locomotion in the peacock was not influenced by a higher rate of muscle force development (Kram & Taylor, 1990; Roberts et al., 1998). The lack of difference in *t*_swing_ between level and incline movement also suggested that the energetic cost of incline locomotion was not influenced by increased swing phase muscle activity to power more rapid foot movements (Heglund & Taylor, 1988; Roberts et al., 1997; Rubenson et al., 2006). When comparing the two inclines, DF did not differ considerably, but 1/*t*_stance_ was lower on a 7° incline than on a 5° incline. The lower rate of force development found at a 7° incline may compensate for some of the elevated cost associated with increasing incline. Here, a lower volume of muscle with slower rates of activity would be recruited, which may reduce the energetic cost of locomotion relative to the gradient ascended (Biewener, 1990; Heglund & Cavagna, 1987).

Although costly compared to level locomotion (Minetti, Ardigo & Saibene, 1994), it has been suggested that changing gait at lower speeds on an incline can afford some metabolic savings (Wickler et al., 2003), which may contribute to the lack of considerable difference in the energetic cost found between the two inclines. However, further analyses of the CoM mechanics and kinematic parameters with increasing *U* and incline are needed to determine whether peacocks change gait at lower speeds with increasing incline. Changing gait also has important physiological implications, such as reducing limb mechanical stress (Farley & Taylor, 1991), thereby allowing longer endurance and reduced risk of injury (Biewener & Taylor, 1986; Smith & Wilson, 2013). As bone and muscle structure is highly conserved (Biewener, 1991), changing to grounded running at lower speeds on an incline may have same effect of reducing peak muscle and bone stress as changing gait with *U* on the level (Perry et al., 1988).

The CoM results indicated variability in the *U* at which gait changes occurred in the peacock on both level ground and at an incline. Although preliminary in comparison with other studies, it has previously been demonstrated that there is overlap in the speeds used for walking, grounded running and aerial running and therefore great variability in the *U* at which a gait transition occurs (Nyakatura et al., 2012). This can be explained by the gradual transitional nature of gait changes in birds, whereby shifts from walking (vaulting) to aerial running gaits occur via vaulting-like and bouncing-like phases (Hancock, Stevens & Biknevicius, 2007). The higher variations seen in phase shifts from walking to running gaits at lower speeds have also been rationalised by the structural and functional locomotor requirements of an avian species. For example, for birds with a crouched posture, maintaining stiff limbs for walking is more costly than adopting a crouched grounded running gait (Nyakatura et al., 2012).

The MCoT values at each incline were considerably lower than the values reported for small animal groups (Farley & Emshwiller, 1996; Full & Tullis, 1990; Lipp, Wolf & Lehmann, 2005; Snyder & Carello, 2008). Similarly, the MCoT value for level movement was lower than the predicted value of 6.62 J kg^−1^ m^−1^ for a 4.6 kg bird (using the equation MCoT = $10.8{M}_{b}^{-0.32}$ Nudds et al., 2010). The MCoT values presented at the intermediate *U*, and the curvilinear relationship shown between MCoT and *U*, were consistent with previous findings in humans (Snyder & Farley, 2011) and horses (Wickler et al., 2000) moving on inclines. The MCoT was also higher on an incline compared to moving on the level, as found in a number of previous investigations (Farley & Emshwiller, 1996; Minetti et al., 2002; Warncke, Bandholtz & Schultze-Motel, 1988; Wunder & Morrison, 1974). The CoL at each incline did not differ considerably, corresponding with the results for net-*P*_met_. The CoL found in both treatments was larger than that predicted (Taylor, Caldwell & Rowntree, 1972), but similar to the $27~\mathrm{J}~{\mathrm{kg}}^{-1}~{\mathrm{m}}_{\mathrm{v}}^{-1}$ found in elk calves (Cohen, Robbins & Davitt, 1978). The efficiencies of converting metabolic energy into mechanical work were also similar between the two inclines, differing from the marked increases in efficiencies (Cohen, Robbins & Davitt, 1978; Lees et al., 2013), and the reduced efficiencies (Full & Tullis, 1990; Taylor, Schmidt-Nielsen & Raab, 1970; White & Yousef, 1978), previously found with increasing incline. However, further comparisons are difficult due to a large number of studies using only one incline gradient. Dissimilarities in other methodologies (e.g., incline gradients used) also highlight the difficulties in finding a consistent pattern of the costs of incline locomotion across species, emphasising the need for future work on incline locomotion.

**Figure 3:**
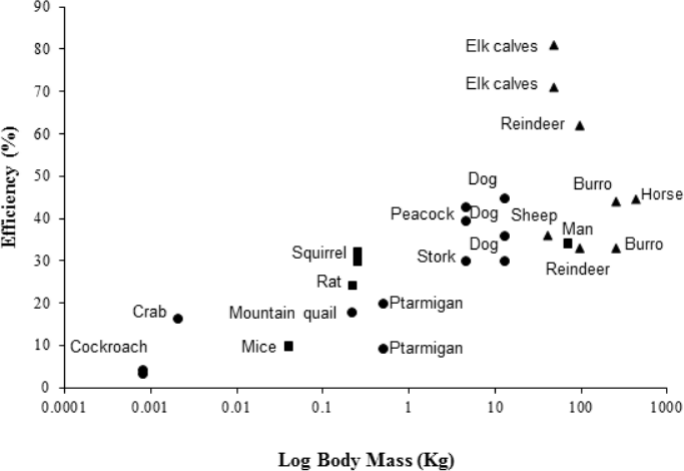
Showing the efficiencies of converting metabolic energy into *mechanical work* in a number of vertebrate (bipedal, depicted by blue markers; quadrupedal, depicted by red markers) and invertebrate (depicted by green markers) animals. For the studied vertebrates, foot posture is also documented (plantigrade, squares; digitigrade, circles; unguligrade, triangles). Inclines range from 2.9°to 90°. Multiple data points at the same body mass indicate the use of more than one incline in the study. Efficiency data was taken from the literature from the following sources: Burro, *Equus esinus* (Yousef, Dill & Freeland, 1972); cockroach, *Periplaneta americana* (Full & Tullis, 1990); dog, *Canis familiaris* (Raab, Eng & Waschler, 1976); elk calves, *Cervus canadensis elsoni* (Cohen, Robbins & Davitt, 1978); ghost crab, *Ocypode quadrata* (Tullis & Andrus, 2011); horse, Equus caballus (Wickler et al., 2000); man, *Homo sapien* (Taylor, Caldwell & Rowntree, 1972); mice, *Mus musculus* (Snyder & Carello, 2008); mountain quail, *Oreortyx pictus* (Snyder & Carello, 2008); ptarmigan, *Lagopus muta hyperborea* (Lees et al., 2013); rat, *Rattus norvegicus* (Snyder & Carello, 2008); reindeer, *Rangifer tarandus groenlandicus* (White & Yousef, 1978); sheep, *Oryes aries* (Clapperton 1964); squirrel, *Tamiasciurus hudsonicus* (Wunder & Morrison, 1974); stork, *Leptoptilus crumeniferous* (Bamford & Maloiy, 1980). Data for the peacock (*Pavo cristatus*) is from the present study. Numerical values and angles of inclination used are provided in supplementary material Table S2.

The peacock has the highest efficiencies, in terms of converting metabolic energy into metabolic power, and lowest CoT values of bipedal birds previously studied (Ellerby et al., 2003; Lees et al., 2013; Snyder & Carello, 2008; Warncke, Bandholtz & Schultze-Motel, 1988), closely followed by the similarly-sized Marabou stork (Bamford & Maloiy, 1980). For comparison with other bipeds and quadrupeds, efficiency data for a number of species are shown in Fig. 3, where larger animals (1–200 kg) tend to have higher efficiencies than smaller animals (>1 kg). However, the relationship between size and efficiency is not linear, suggesting structural and postural differences play an important role in determining the costs and efficiencies of locomotion (Reilly, McElroy & Biknevicius, 2007). For example, being larger is associated with longer stride lengths and lower stride frequencies (Heglund et al., 1982; Heglund & Taylor, 1988; Kram & Taylor, 1990; Taylor, Heglund & Maloiy, 1982). Larger animals also tend to have straighter limbs, longer tendons with a smaller cross-sectional area compared to the muscles, and ungulate or digitigrade foot placement. Therefore, larger animals have less crouched locomotor positions, increased elastic potential energy savings and require lower force generation per unit mass than smaller, more crouched animals (Biewener, 1990; Pontzer, 2005; Reilly, McElroy & Biknevicius, 2007).

In summary, we have found that the energetic cost of locomotion is significantly higher on an incline than on the level ground, with changes in DF and 1/*t*_stance_ corresponding with the energetic results. Interestingly, the peacock had the highest efficiencies of locomotion of any bipedal bird studied, yet no other kinematic changes were observed with increasing incline. Taken together, our results accentuate the importance of combining energetic and kinematic responses, while also acknowledging allometric and phylogenetic differences, to gain a clearer understanding of the intrinsic costs associated with incline locomotion across the animal kingdom.

## Supplemental Information

10.7717/peerj.987/supp-1 Supplemental Information 1 Raw data files.

10.7717/peerj.987/supp-2 Table S1 and S2 Table S1. The peacock energetic results showing the number of individuals (*N*) in each treatment (speed and incline) including body mass (± standard deviation). Net-*P*_met_ refers to metabolic power consumption minus resting metabolic rate. CoT is the cost of transport at each speed and CoL refers to the cost of lifting at each incline (5°and 7°). Efficiencies (of converting metabolic energy into metabolic power for movement) are also shown.
Table S2. The efficiency of converting metabolic energy into mechanical work for a number of species, as plotted in Fig. 3. Data was collected from the literature. Angles of inclination used in each study are also provided.
