# Supplementary material for: The metabolic cost of walking on an incline in the Peacock (Pavo cristatus)
Source: PeerJ. 2015 Jun 2;3:e987. doi: 10.7717/peerj.987 (PMC4458126; doi:10.7717/peerj.987)
Supplement: Table S1 and S2 — Table S1. The peacock energetic results showing the number of individuals (N) in each treatment (speed and incline) including body mass (± standard deviation). Net-Pmet refers to metabolic power consumption minus resting metabolic rate. CoT is the cost of transport at each speed and CoL refers to the cost of lifting at each incline (5°and 7°). Efficiencies (of converting metabolic energy into metabolic power for movement) are also shown. Table S2. The efficiency of converting metabolic energy into mechanical work for a number of species, as plotted in Fig. 3. Data was collected from the literature. Angles of inclination used in each study are also provided. [file peerj-03-987-s002.docx]

Table S1. The peacock energetic results showing the number of individuals (*N*) in each treatment (speed and incline) including body mass (+/- standard deviation). Net-*P*_met_ refers to metabolic power consumption minus resting metabolic rate. CoT is the cost of transport at each speed and CoL refers to the cost of lifting at each incline (5⁰ and 7⁰). Efficiencies (of converting metabolic energy into metabolic power for movement) are also shown.

| **Speed**  **(m s^-1^)** | **Incline (degrees)** | ***N*** | **Body Mass (kg)** | **Net Pmet (W kg)** | **CoT (J kg^-1^ m^-1^)** | **CoL (J kg^-1^ m_v_^-1^)** | **Efficiency (%)** |
| --- | --- | --- | --- | --- | --- | --- | --- |
| 0 | 0 | 6 | 4.67+/-0.14 |  |  |  |  |
| 0.5 |  | 6 |  | 3.16 | 6.33 |  |  |
| 0.75 |  | 6 |  | 4.14 | 5.52 |  |  |
| 1 |  | 5 | 4.7+/-0.17 | 5.65 | 5.65 |  |  |
| 0 | 5 | 6 | 4.58+/-0.14 |  |  | 22.84 | 42.9 |
| 0.5 |  | 6 |  | 4.46 | 8.91 |  |  |
| 0.75 |  | 6 |  | 5.63 | 7.51 |  |  |
| 1 |  | 5 | 4.52+/-0.16 | 8.9 | 8.9 |  |  |
| 0 | 7 | 6 | 4.56+/-0.16 |  |  | 24.79 | 39.53 |
| 0.5 |  | 6 |  | 4.37 | 8.74 |  |  |
| 0.75 |  | 6 |  | 6.83 | 6.83 |  |  |
| 1 |  | 4 | 4.74+/-0.14 | 8.54 | 8.54 |  |  |

Table S2. The efficiency of converting metabolic energy into mechanical work for a number of species, as plotted in Fig. 3. Data was collected from the literature. Angles of inclination used in each study are also provided.

| **Species** | **Efficiency** | **Body Mass** | **Angle** | **References** |
| --- | --- | --- | --- | --- |
| Cockroach (*Periplaneta americana*) | 4.4 | 0.0008 | 45 | (Full and Tullis, 1990) |
|  | 3.4 | 0.0008 | 90 |  |
| Ghost crab (*Ocypode quadrata*) | 16.5 | 0.002 | 20 | (Tullis and Andrus, 2011) |
| Mice (*Mus musculus*) | 9.9 | 0.04 | 10 | (Snyder and Carello, 2008) |
| Guinea fowl (*Numida meleagris*) | 8 | 1.52 | 6 | (Ellerby et al., 2003) |
|  | 12 | 1.52 | 11 |  |
| Mountain quail (*Oreortyx pictus*) | 17.9 | 0.21 | 10 | (Snyder and Carello, 2008) |
| Rats (*Rattus norvegicus*) | 24.2 | 0.22 | 10 | (Snyder and Carello, 2008) |
| Red squirrel (*Tamiasciurus hudsonicus*) | 30 | 0.252 | 6 | (Wunder and Morrison, 1974) |
|  | 32 | 0.252 | 18 |  |
| Svalbard ptarmigan (*Lagopus muta hyperborea*) | 9.5 | 0.49 | 4.2 | (Lees et al., 2013) |
|  | 20.1 | 0.49 | 7.4 |  |
| Marabou stork (*Leptoptilus crumeniferus*) | 30 | 4.5 | 11 | (Bamford and Maloiy, 1980) |
| Indian peacock (*Pavo cristatus*) | 42.9 | 4.5 | 5 | Current study |
|  | 39.5 | 4.5 | 7 |  |
| Dog (*Canis familiaris*) | 30 | 12.8 | 11.5 | (Raab et al., 1976) |
|  | 36 | 12.8 | 6.7 |  |
| Sheep (*Oryes aries*) | 45 | 12.8 | 4.2 | (Clapperton, 1964) |
|  | 36 | 40.8 | 5.12 |  |
| Elk Calves (*Cervus canadensis nelson*) | 81 | 48 | 11.7 | (Cohen et al., 1978) |
|  | 71 | 48 | 4.2 |  |
| Man (*Homo sapien*) | 34 | 70 | 15 | (Taylor et al., 1972) |
| Reindeer (*Rangifer tarandus groenlandicus*) | 33 | 96 | 5.1 | (White and Yousef, 1978) |
|  | 62 | 96 | 2.9 |  |
| Burro (*Equus asinus*) | 33 | 253.5 | 9.6 | (Yousef et al., 1972) |
|  | 44 | 253.5 | 5.7 |  |
| Horse (*Equus caballus*) | 44.56 | 431 | 0.1 | (Wickler et al., 2000) |
